# Frailty index, mortality, and length of stay in a geriatric short-stay unit in Guadeloupe

**DOI:** 10.3389/fmed.2022.963687

**Published:** 2022-08-12

**Authors:** Larissa Vainqueur, Nadine Simo-Tabue, Roxane Villeneuve, Dorice Dagonia, Bernard Bhakkan-Mambir, Ludwig Mounsamy, Vaynome Delacroix, Maturin Tabue-Teguo

**Affiliations:** ^1^CHU de Guadeloupe, Abymes, Guadeloupe; ^2^Equipe ACTIVE, INSERM 1219, Université de Bordeaux, Bordeaux, France; ^3^CHU de Martinique, Pôle de Gériatrie, Fort-de-France, Martinique; ^4^Equipe LAMIA, Université des Antilles, Pointe-á-pitre, Guadeloupe

**Keywords:** frailty, mortality, SARS-CoV-2, in-hospital, length of stay (LHS)

## Abstract

**Context:**

The COVID-19 pandemic has placed a tremendous stress on healthcare systems and caused reorganization. As the pandemic intensifies, identifying the profile of patients with COVID-19 was primordial in order to predict negative outcomes and organize healthcare resources. Age is associated with COVID-19’s mortality, but for obvious ethical reasons, chronological age cannot be the sole criterion for predicting negative outcomes.

**Objective:**

The objective of this study was to determine the relationship between frailty index (FI) and length of hospital stay, and death in a non-COVID population of patients aged 75 years old and above.

**Methods and design:**

A retrospective, analytical, single-centered observational study was performed in the geriatric short-stay accommodation unit at Guadeloupe University Hospital. For this study, 158 patients who were at least 75 years old were recruited from November 2020 to May 2021. FI was calculated as the number of deficits in a participant divided by the total number of deficits considered (the cut-off of FI is.25 in outpatient). Multivariate logistics regression analyses were conducted to assess the association between frailty and death, and length of stay.

**Results:**

The average age of the participants was 85.7 ± 6.74 with a range of 75–104. Twenty-four of the patients died during hospitalization. FI was only significantly associated with mortality even after adjustment for age and gender (HR 26.3, 95% CI 1.7–413.4, *P* = 0.021). The association was stronger in the highest tertile of the FI (age- and gender-adjusted HR 4.6, 95% CI 1.39–15.11, *P* = 0.01). There was no significant interaction between FI and length of stay.

**Conclusion:**

Our study shows an association between FI (in terms of age-related deficit accumulation) and mortality in a non-COVID geriatric short-stay unit in Guadeloupe. The FI seems to have a lower capacity to catch events such as length of stay in this very complex population. Further research studies have to be conducted for better understanding and investigation of our findings.

## Introduction

The number of people aged 65 and over has dramatically increased over the last several decades (1). From 461 million in 2004, they could reach 2 billion by 2050 (2). In Guadeloupe, they could represent 25% of the population within the next 10 years (3). As a geriatric syndrome, frailty is a long process resulting from the loss of capacity reserve in multiple physiological systems. It results from loss of resources at multiple levels and strongly predicts adverse health outcomes such as hospitalization, disability, and death (4). The operationalization of the concept of frailty is complex, ranging from physical frailty alone to a more comprehensive assessment of comorbidities, falls, and physical factors (5). Rockwood defines frailty as the accumulation of physical and psychological deficits (6). Based on this model, he developed the frailty index, which takes into account clinical signs, geriatrics syndromes, and level of disability (7). Using this index, patients can be identified as frail or robust, a helpful categorization for patient management.

Since 2019, the COVID-19 pandemic has been affecting millions of people around the world (8). The global spread of COVID-19 increased the number of scientific publications on the profile of patients with COVID19 (9). Although it appears to affect all age groups, older, frail people are at greater risk of adverse events following contamination with SARS-CoV-2 (10). Several studies showed that age is associated with COVID-19 mortality (11, 12). People over 75 years old account for more than 70% of COVID-related deaths in France (13). For obvious ethical reasons, chronological age cannot be the sole criterion for predicting negative outcomes (14).

Moreover, age and comorbidities can have an influence on the severity of the disease and are likely to impact the length of stay (15). Predicting the length of stay and bed demand can provide information for better patient care.

Guadeloupe (French West Indies area), like the rest of France and many other countries, has taken unprecedented measures to prevent the virus from spreading and to protect older, frail, and dependent older people, such as quarantine, lockdown, and cancellation of non-urgent consultations. These measures led to complete reorganization of the healthcare system for people over 70 years old. Guadeloupe has recently faced a deadly outbreak, resulting in high rates of hospitalization and death among older people, in a complex context of low vaccination rates and distrust in its medical and political institutions. Clinicians are, more than ever, in need of validated instruments to assess their patients’ risks of adverse health outcomes.

The objective of this study was to determine the relationship between the frailty index and length of hospital stay, and mortality in a non-COVID population of patients over 75 years old using data from a short-stay accommodation unit for elderly people in the Guadeloupe University Hospital.

## Materials and methods

### Study sample

A retrospective, analytical, single-centered observational study was performed in the geriatric short stay accommodation unit at Guadeloupe University Hospital.

From November 2020 to May 2021, 158 patients who were at least 75 years old were recruited in the geriatric short-stay accommodation unit of the Guadeloupe University Hospital.

The geriatric unit in our hospital and in France in general only admits patients aged 75 years and older. A local criterion for admitting patients in geriatrics facilities is age of 75 years or older with multiple comorbidities.

During this period, there were two units for short stay: a COVID-free short stay one and a unit with patients with COVID-19. We decided to include only patients who were in the COVID-free unit in order to explore the frail syndrome in its totality in the context of the pandemic.

Every patient in this unit was invited to participate. People who expressed their opposition were not recruited.

Patients excluded are those who expressed their objections after receiving the study information letter. Moreover, patients with severe neurocognitive disorders and behavioral disorders could not be included, since we did not have all the data to build the frailty index. The data were collected from medical charts of the hospital and included sociodemographic (age and gender) and lifestyle characteristics, chronic diseases, and functional status.

The Ethical Committee of the Guadeloupe University Hospital approved the study protocol.

All the participants and their proxies were informed by the study investigators about the research and left free to accept or refuse to participate.

### The frailty index

The frailty index is a cumulative index that relies on available variables in a given dataset, with a set of 40 variables. The variables may include diseases, laboratory abnormalities, cognitive impairments, and disabilities in (instrumental) activities of daily living. The score is calculated as the number of deficits in a participant divided by the total number of deficits considered. In this study, 34 variables were available in the participants’ medical records (Table 1). Each variable is coded “0” if absent and “1” if present. Items included current diseases, ability to perform activities of daily living, nutritional status, and cognitive status. No variable had more than 5% missing data (16).

**TABLE 1:** List of variables and cut point used for the Rockwood index.

List of variables used for the frailty index	Cut point
Cardiovascular diseases	Yes = 1, No = 0
Vascular diseases	Yes = 1, No = 0
Endocrine diseases	Yes = 1, No = 0
Neurological diseases	Yes = 1, No = 0
Psychiatric disorders	Yes = 1, No = 0
Kidney and urinary disorders	Yes = 1, No = 0
Gastrointestinal diseases	Yes = 1, No = 0
Osteoarticular diseases	Yes = 1, No = 0
ENT disorders	Yes = 1, No = 0
Cancer and hematological diseases	Yes = 1, No = 0
Vitamine D deficiency	<10 ng/ml = 1, 11–29 ng/ml = 0.5, >30 ng/ml = 0
MMSE	<10 = 1, 11–17 = 0.75, 18–20 = 0.5, 20–24 = 0.25, >24 = 0
>1 fall in the last 12 month	Yes = 1, No = 0
Regular medical visit	Yes = 1, No = 0
Help Bathing	Yes = 1, No = 0
Help Dressing	Yes = 1, No = 0
Help Using Toilet	Yes = 1, No = 0
Incontinence	Yes = 1, No = 0
Help Eating	Yes = 1, No = 0
Help Walking around house	Yes = 1, No = 0
Ability to use telephone	Yes = 1, No = 0
Help shopping	Yes = 1, No = 0
Help with meal preparation	Yes = 1, No = 0
Help with Housework	Yes = 1, No = 0
Help with laundry	Yes = 1, No = 0
Ability to use transportation	Yes = 1, No = 0
Help taking medication	Yes = 1, No = 0
Help with finances	Yes = 1, No = 0
Albumine levels	<35 g/L = 1, 35-40 g/L = 0.5, > 40 g/l = 0
Far vision	Yes = 1, No = 0
Near vision	Yes = 1, No = 0
Eye diseases	Yes = 1, No = 0
MNA	<17 = 1, 17-23,5 = 0.5, > 24 = 0
Obesity	Yes = 1, No = 0

MNA, Mini Nutritional Assessment; MMSE, Mini-Mental State Examination.

### Outcome

The outcomes of interest for the present analysis were mortality and length of hospital stay.

Length of stay was calculated as the number of days spent in the non-COVID geriatric unit by a patient (including patients who were transferred to the geriatric unit and came back or died after few hours spent in the unit).

Mortality during the stay was determined from medical charts and administrative documentations.

### Other variables

Sociodemographic information included age, gender, and way of life. Physical functions were determined using the basic activities of daily living (ADL) and defined using the Katz scale (17), and the modified instrumental ADL (IADL) (18).

### Statistical analysis

For statistical analysis of the data, quantitative variables were expressed as means ± standard deviation (SD) and qualitative variables as number of participants and percentages.

We conducted univariate and multivariate logistic regression analyses to assess the association between frailty and death, and length of stay. We also tested interactions between the FI and length of stay. Statistical significance was set at a *P* of less than.05. All the statistical analyses were performed using the R-Studio statistical software.

## Results

The descriptive characteristics of the sample (n = 158) are presented in Table 2.

**TABLE 2:** Characteristics of patients according to death events.

	Sample	Death events		*P*
	N = 158	No N = 134	Yes N = 24	
Age (year)	85.70 ± 6.74	85.70 ± 6.81	85.70 ± 6.44	0.82
Gender (women)	81	68 (51%)	9 (38%)	0.23
ADL limitations, 0–6	3.6 ± 2.13	3.9 ± 2.08	2.125 ± 1.74	**<0.001**
IADL limitations, 0–4	2.7 ± 1.44	2.62 ± 1.42	3.1 ± 1.46	**0.041**
Live at home	96 (60.8%)	130 (97%)	22 (92%)	0.22
Cardiovascular diseases	125 (79.11%)	106 (74%)	19 (79%)	0.99
Neurological diseases	81 (50.3%)	70 (52%)	11 (45%)	0.56
Endocrine disease	68 (40.03%)	58 (43%)	10 (41%)	0.88
Kidney and urinary disorders	47(29.75%)	37 (28%)	10 (41%)	0.17
Frailty index	0.42 ± 0.18	0.41 ± 0.17	0.47 ± 0.18	**0.011**
MMSE score (/30)	13.2 ± 8.9	13.87 ± 8.76	8.71 ± 9.35	**0.014**
Length of stay (days)	11.9 ± 6.72	12.54 ± 6.84	11.7 ± 5.29	**<0.01**
Death events	24 (15.2%)	–	–	–

The results are presented as means ± SDs or percentages. FI: frailty index, ADL: activities of daily living.

IADL, instrumental ADL; MMSE, Mini-Mental State Examination.

The average age of the participants was 85.7 years ± 6.74, with a range of 75-104. Half of the subjects (49%) were female. The average length of stay was 11.9 ± 6.72. A total of 24 patients died during hospitalization in the geriatric short-stay unit. The three main reasons of hospitalization were heart failure, stroke, and confusion. Compared to survivor participants, residents who died were more dependent (2.125 ± 1.74, *P* < 0.001), had lower cognitive performance, and had no significant differences in terms of cardiovascular, neurological, and endocrine diseases. Regarding the variables of interest for this study, the results show significant differences between participants who died and survivors for FI (0.47 ± 0.18 vs.41 ± 0.17, *P*:0.011).

The mean of the FI for the whole sample was.42 (SD.18) with a median of.41 (range.03–0.9). Table 3 shows the relationship between the FI and mortality. The FI (as a continuous variable) was significantly associated with mortality even after adjustment for age and gender [HR: 26.3 (1.7–413.4), *P* = 0.02]. The risk of death was higher in participants in the highest tertile of the FI even after adjustment of age and gender [HR:4.6 (1.39–15.11), *P* = 0.01]. We also assessed the relationship between the FI and length of hospital stay (Table 4). The FI (as a continuous variable) and after stratification by FI-tertile was not significantly associated with length of stay (*P* = 0.16). No significant interaction of age and/or gender was reported in the studied relationship between the FI and length of stay (*P* = 0.683).

**TABLE 3:** Relationship between frailty index and mortality.

	Unadjusted HR (95% CI)	*P*	Adjusted* HR (95% CI)	*P*
Frailty Index (continuous),	25.3 (1.64–389.5)	0.021	26.3 (1.7–413.4)	**0.020**
**Frailty index tertiles**				
Tertile 1 (FI: 0.00–0.40)	1		1	
Tertile 2 (FI: 0.41–0.58)	1.20(0.30–4.73)	0.79	1.17 (0.29–4.7)	0.82
Tertile 3 (FI: 0.59–1.00)	4.63 (1.42–15.13)	0.01	4.60 (1.39–15.11)	**0.01**

*Adjusted for age and gender. FI, frailty index; CI, confidence interval; P, p-values; HR, Hazard ratio,

**TABLE 4:** Relationship between frailty index and length of stay.

	Unadjusted HR (95% CI)	*P*	Adjusted* HR (95% CI)	*P*
Frailty Index (continuous),	0.02 (0.0005–6.49)	0.18	0.01 (0.003–5.6)	0.16
**Frailty index tertiles**				
Tertile 1 (FI: 0.00–0.40)	1		1	
Tertile 2 (FI: 0.41–0.58)	5.68 (0.42–75.43)	0.18	5.36 (0.39–72.92)	0.21
Tertile 3 (FI: 0.59–1.00)	0.28 (0.02–3.84)	0.34	0.26(0.02–3.58)	0.64

*Adjusted for age and gender. FI, frailty Index; CI, confidence interval; P, p-values; HR, hazard ratio.

As part of an additional analysis on the annual report of geriatric unit activities (160 patients), we have shown that the average of FI was.32 (SD:0.28), death rate was 10.3%, length of stay was 8.6 (SD: 4.3), and MMSE score was 16.2 (SD: 7.3) over the same period 1 year before the COVID-19 pandemic.

## Discussion

Based on an in-hospital-based sample, our study shows that the FI is a predictor of mortality. The associations were independent of age and gender. The results are consistent with other studies on relationship between the FI and mortality. Rockwood et al. in a nursing home (19) in Canada, and Xiaowei Song al (20), in a general population, proved that the Rockwood Index was associated with mortality.

However, the association in this survey must be interpreted with caution because of the characteristics of this sample [the mean of FI (0.41) was higher than what was expected on a traditional geriatric unit (0.32) 1 year before the COVID-19 pandemic]. The patients in this study were recruited during the COVID-19 pandemic. The non-COVID geriatric unit would exclusively accept patient coming from the emergency room. We dealt with a very dependent and frail population. The population was homogeneous with a lot of deficits. Global emergency implied a total reorganization of the health system (21). The lockdown led to decrease or cease most non-urgent COVID-19 activities. Physical examinations were reduced, and this situation may have led to delay in diagnosis of patients without COVID-19 (22), not to mention many patients with severe diseases delayed their medical consultations because they thought healthcare units were not accessible (23, 24). The recruitment of the short stay unit has changed in the current context of the pandemic. It seems that the predictive capacity of the index might not be optimal for people with so many deficits.

In addition, we found that the FI is not associated with length of stay in this survey. This result is not consistent with other studies in the scientific literature (25, 26). Length of stay is a measure of effectiveness and an economic performance indicator; however, it is not always a quality indicator, because it cannot predict a positive or a clinical outcome. A study shows that length of stay varies with post-hospitalization destination. Patients who return home had shorter length of stay (27).

Several reason may explain our results. In this study, the average length of stay is higher than usual in this type of unit. Moreover, the average frailty index is.41, and it is way above the.25 cuff-off. This average FI score is similar to the cut-off found in Tabue-Teguo et al. for people who lived in a nursing-home (28) and Wallace and al. for people with Alzheimer’s disease (29). The population in this study had many deficits (FI average 0.41) and was closer to the outcome (mortality) than a community dwelling population.

This means that there is a ceiling effect, and that the predictive capacity of the index might decrease for people with many deficits. This result shows that this population is highly dependent. Unlike the usual population hospitalized in the short-stay unit, this result highlights the seriousness of the medical condition of our population and confirms the delay in the care of non-COVID subjects during this pandemic.

Our study confirms the capacity of FI to predict survival in every context and independent of confounders.

This study is the first one in the Caribbean region on this subject. All the patients hospitalized during the study period were included. Although the study is retrospective, the quality of the data collected in the medical files makes it possible to capture geriatric syndromes. This study is exploratory and has limits. The main weakness of our study is that the sample is not representative of the geriatric population in the Caribbean area, and it has a small size. There is a selection bias, because we choose to include only patients in the non-COVID geriatric short-stay unit. This study is retrospective; it is not representative of the in-hospital population. In addition, it is a longitudinal, prognostic but observational study that does not allow for demonstration of a causal link. Given the place of the study, the results cannot be extrapolated to patients outside of the hospital or nursing homes and foster families. The competing risk (death) could be a potential limitation in interpreting the absence of association between the FI and length of stay.

Our result suggests a negative health impact of the COVID-19 pandemic on older people without SARS-CoV-2 infection.

## Conclusion

Our study shows an association between the FI (in terms of age-related deficit accumulation) and mortality in a non-COVID geriatric short-stay unit in Guadeloupe.

The FI may be considered as a simple tool to measure the frailty of an individual and exposure to mortality. Nevertheless, the FI seems to have a lower capacity to catch events such as length of stay in this very complex population compared with what is shown in community-dwelling elders. Further research has to be conducted for better understanding and investigation of our findings.

## Data availability statement

The raw data supporting the conclusions of this article will be made available by the authors, without undue reservation.

## Author contributions

LV and NS-T conceptualized and designed the study, interpreted the data, wrote the manuscript, had full access to all of the data in the study, took responsibility for the integrity of the data, and the accuracy of the data analysis. RV, BB-M, NS-T, and MT-T contributed to statistical analyses, interpretation of the data, and drafting of the manuscript. DD and LM contributed to interpretation of the data and revision of the manuscript. MT-T contributed to conceptualization and design, interpretation of the data, and drafting of the manuscript, and had full access to the study data. All authors contributed to the article and approved the submitted version.
